# Clinical Evaluation of the Effects of Topical Indocyanine-green Mediated Photosensitiser vs Aloe Vera Gel as Adjunct Therapy to Scaling and Root Planing in Chronic Periodontitis Patients

**DOI:** 10.3290/j.ohpd.b2082037

**Published:** 2021-09-30

**Authors:** Zeeshan Qamar, Salah Abdalkreem Almohana, Abdulmohsen Khalid Alanazi, Abdulelah Khalid Alanazi, Alhanouf Abdulkarem Almohana, Tayyaba Zeeshan

**Affiliations:** a Assistant Professor, Department of OMFS and Diagnostic Sciences, Faculty of Dentistry, Riyadh Elm University, Riyadh, Saudi Arabia; Department of Oral Biology, Liaquat College of Medicine and Dentistry, Karachi, Pakistan. Principal investigator, performed treatment, wrote the manuscript.; b Dentist, Department of OMFS and Diagnostic Sciences, Faculty of Dentistry, Riyadh Elm University, Riyadh, Saudi Arabia. Treated patients, co-wrote the manuscript.; c Dentist, Department of OMFS and Diagnostic Sciences, Faculty of Dentistry, Riyadh Elm University, Riyadh, Saudi Arabia. Played vital role in treating patients, co-wrote the manuscript.; d Dentist, Department of Oral and Craniofacial Sciences, University Malaya, Malaysia. Statistical analysis, treated patients, co-wrote the manuscript.

**Keywords:** aloe vera, chronic periodontitis, photodynamic therapy, scaling and root planing

## Abstract

**Purpose:**

To clinically evaluate and compare the efficacy of indocyanine green mediated photodynamic therapy (PDT) and aloe vera (AV) gel when used as adjunct therapy to scaling and root planing (SRP) for treatment of chronic periodontitis.

**Materials and Methods:**

One hundred fifty patients were randomly assigned to three treatment groups: group 1 (SRP), group 2 (SRP+PDT) and group 3 (SRP+AV). Four clinical parameters – plaque index (PI), bleeding on probing (BoP), periodontal (PD) pocket depth and clinical attachment level (CAL) – were evaluated at baseline and 3 and 6 months post treatment. Additionally, the amount of three inflammatory – cytokines IL-6, IL-8 and TNF-α – in gingival crevicular fluid (GCF) was identified using an enzyme-linked immune-sorbent assay (ELISA).

**Result:**

Statistically significant improvement was observed for all clinical parameters in group 3 at follow-up in comparison to groups 1 and 2. Individuals treated with adjunct PDT showed statistically significant reduction in moderate (4-5 mm) and deep (≥6 mm) PD pockets at the 3-month follow-up. Group-2 and -3 patients displayed statistically significant reductions in cytokines IL-6, IL-8 and TNF-α at the 3-month follow-up in comparison to group 1 patients. This reduction in cytokines was maintained at the 6-month follow-up.

**Conclusion:**

Adjunct treatment regimens PDT and AV gel statistically significantly contributed to decreasing inflammation in periodontal tissue. AV /gel showed potential to decrease BoP, whereas PDT can facilitate increasing the clinical attachment level.

Periodontitis, a common inflammatory disease of the oral cavity, occurs as a result of interaction between microbial biofilm and host immune response.^16,24,40^ Early stage gingival inflammation, gingivitis, may progress into severe periodontal (PD infection) characterised by destruction of alveolar bone, loss of clinical attachment and deep PD pockets, if left untreated.^40^ The prolonged colonisation of PD pockets with gram negative microbes actively initiates immune-inflammatory response leading to chronic periodontitis.^38,41^ Additionally, habits (smoking) and systemic diseases may contribute to PD disease.^19,28,44^ The basic treatment for chronic periodontitis is to reduce the microbial load at the infected site by scaling and root planing (SRP).^14^ The effectiveness of SRP is reduced in misaligned teeth and with moderate to severe PD pocket depths, as removal of microbes at the infected site is incomplete.^2^ In order to overcome the limitations of SRP, various adjunct surgical and non-surgical treatments have been introduced, including open-flap microbial removal, laser,^11^ antibiotics (eg, azithromycin and clarithromycin)^1,34^ and bisphosphonates.^3,4^

As an alternative to chemical medications, various natural/herbal medicines with minimal side effects have been studied as an adjunct to SRP for treatment of PD infections. These include green tea, *Salvadora persica* and *Mikania laevigata*.^10,21,22,39^ Aloe vera (AV) is in the Liliaceae plant family. Various studies have shown a medicinal effect of AV for the treatment of inflammation, arthritis and microbial infections.^20,43^ Additionally, researchers have evaluated the effect of AV gel on systemic and non-systemic diseases including cancer and aphthous stomatitis, and AV’s antimicrobial potential when used as an active ingredient in toothpastes and mouthwashes for treatment of candida and gingival infections.^8,11,13,29,33^

Photodynamic therapy (PDT), being a minimally invasive technique, is currently suggested as the treatment of choice for various disorders of the human body.^35^ PDT mainly comprises two components: photosensitisers and dental-unit light of a particular wavelength.^36^ Dental-unit light of specified strength interact with the photosensitiser and help produce reactive oxygen species (ROS). The ROS interact with various molecules in the intracellular matrix, leading to cell death.^23,25^

Topical application of natural medications in dentistry is thought to improve conditions like periodontitis. Thus the main aim of the current study was to compare the effect of topical PDT and AV gel as an adjunct therapy to SRP in chronic periodontitis patients with moderate to deep PD pockets.

## Materials and Methods

With prior ethical approval from the ethics boards of Riyadh Elm University and Liaquat College of Medicine and Dentistry, a 6-month randomised controlled trial (RCT) was conducted on systemically healthy patients. Patients who met the following three criteria were included in the study: 1. age ≥ 30 years; 2. ≥ 20 natural teeth; and 3. chronic periodontitis (≥ 3 mm CAL and PD pocket depth on at least 30% of dental sites).^41^ A total of 150 patients were included in the study after obtaining written consent at the dental hospital. Individuals with chronic systemic diseases, had PD therapy during last 6 months, habitually used chewable and non-chewable tobacco (smoking), consumed alcohol, or used certain medications during last three months (steroidal and non-steroidal drugs, antibiotics, antidepressants) were excluded from the study.

The included patients were randomly divided into three treatment groups: group 1, SRP (n = 50); group 2, PDT + SRP (n = 50); group 3, AV gel + SRP (n = 50) respectively.

Three parameters (PI, PD pocket depth, BoP and CAL) were recorded for all three groups at different time points – baseline, 3 and 6 months – at six different sites (mesio-buccal, mid-buccal, disto-buccal, mesio-lingual, mid-lingual and disto-lingual) of all teeth except for the wisdom teeth using a graded periodontal probe. Inter- and intra-examiner reliability was Kappa=0.94 and 0.87, respectively.

### PBS Solution

PBS solution was prepared according to the manufacturer’s instructions (Oxoid; Basingstoke, UK). Later, the pH of the PBS solution was adjusted to 7.0 by dropwise addition of sodium hydroxide (NaOH). The solution was kept at 23°C until further use.

### GCF Interleukin and Necrotising Factor Measurement

GCF samples were collected from all 150 patients at baseline, 3 and 6 months to determine the level of three cytokines: interleukin-6 (IL-6); interleukin-8 (IL-8); and tumor necrosis factor-alpha (TNF-α). Prior to collecting GCF using paper strips, supragingival plaque was removed from the respective sites. The paper strips were inserted in the supragingival pockets for a minimum of 15 s. Later, the strips were stored in 300 ml of PBS solution. A Periotron 8000 (Oraflow; Smithtown, NY, USA) was used for GCF analysis. ELISA kits (Elisa Plate Reader, BioTek Instruments; Winooski, VT, USA) were used according to manufacturers’ instructions to measure IL-6, IL-8 and TNF-α levels.

### AV Gel

Fresh AV leaves were thoroughly rinsed with deionized water. Then, the leaves were sectioned with a knife and the gelatinous AV pulp was removed with a sterile spatula and collected in a sterile container. The bottle was refrigerated at 0°C until further use.

### Treatment Protocols

The treatment protocols followed for the three groups were:

- Group 1: Single-session SRP with full-mouth ultrasonic scaling by qualified dentists using hand curettes under local anesthesia. Later, patients were instructed in brushing and flossing techniques with appropriate oral hygiene instructions. They were also prescribed 0.12% chlorhexidine gluconate (CHX) mouthrinse twice daily for one month.
- Group 2: After single-session full-mouth SRP, the photosensitiser indocyanine green (ICG) was applied in PD pockets with a blunt needle for 2 min followed by photo-activation with a GaAIAs laser diode (AMD Lasers; Indianapolis, IN, USA) of 810 nm wavelength at 100 mW. Subsequently, the ICG was rinsed off with normal saline solution.
- Group 3: After full-mouth SRP, the AV gel was introduced into PD pockets with a syringe. The respective sites were covered by cotton rolls for 1 h. Patients were instructed to avoiding eating hard/sticky foods for a minimum of 1 h.

Statistical analysis was carried out using SPSS software (v 19, IBM; Armonk, NY, USA). The Kruskal-Wallis test was used to compare the changes in clinical parameters at three different time points in within and between treatment groups, whereas Bonferroni’s post-hoc test was applied for serial comparisons.

## Results

The demographic and baseline clinical characteristics were recorded as shown in Table 1. A total of 150 patients participated in the study, with n = 50 per treatment group. A higher number of male participants were observed in all three treatment groups. The mean age of the patients in groups 1, 2, and 3 was 46.76 ± 8.2, 48.34 ± 6.7 and 51.02 ± 9.4, respectively. Moderate PD pockets for patients in groups 1, 2 and 3 measured 5.08 mm ± 0.58, 4.90 mm ± 0.61, and 4.97 mm ± 0.48, respectively, with no statistically significant difference. Similarly, deep periodontal pockets in groups 1, 2 and 3 also did not differ statistically significantly. Furthermore, there was no statistically significant difference between clinical baseline measurements for CAL in the three treatment groups within moderate pockets and deep pockets.

**Table 1:** Sociodemographic and baseline clinical variables

Treatment groups	Gender		Age ± SD (years)	PI ± SD (%)	BoP ± SD (%)	PD pockets ± SD (mm)		CAL ± SD (mm)	
	Males	Females				Moderate pockets (4−5 mm)	Deep pockets (≥6 mm)	Moderate pockets (4−5 mm)	Deep pockets (≥6 mm)
Group 1 (SRP) n = 50	31	19	46.76 ± 8.2	37.48 ± 6.9	38.43 ± 7.3	5.08 ± 0.58	6.32 ± 0.77	4.89 ± 0.93	6.67 ± 0.71
Group 2 (SRP+PDT) n = 50	28	22	48.34 ± 6.7	33.86 ± 7.1	40.19 ± 8.2	4.90 ± 0.61	6.41 ± 1.03	5.44 ± 0.93	6.83 ± 1.03
Group 3 (SPR+AV gel) n = 50	33	17	51.02 ± 9.4	35.76 ± 5.59	36.32 ± 4.9	4.97 ± 0.48	6.19 ± 1.26	4.57 ± 0.69	6.91 ± 1.08

Four clinical parameters – PI, BoP, depth of PD pockets and CAL – were evaluated at baseline, 3 and 6 months, as shown in Table 2. At baseline, there was no statistically significant difference between the four clinical parameters in the three treatment groups. A statistically significant difference for clinical parameter PI was observed at the 3- and 6-month follow-ups between group 1 and groups 2/3 (p < 0.05), but the difference between groups 2 and 3 was insignificant. A statistically significant improvement in BoP was observed after treatment with SRP+ PDT (group 2) and SRP+AV gel (group 3). Improvement in CAL and a decrease in PD pocket depth was observed at 3- and 6-months follow-up for all three treatment groups. The baseline values for clinical parameters were statistically significantly improved at the 3- and 6-month follow-ups (p < 0.05), as shown in Table 2.

**Table 2:** Changes in clinical parameters in three treatment groups at three time points: baseline, 3 months, and 6 months

							PD pockets ± SD (mm)			PD pockets ± SD (mm)			CAL ± SD (mm)					
	PI ± SD (%)			BoP ± SD (%)			Moderate pockets (4−5 mm)			Deep pockets (≥6 mm)			Moderate pockets (4−5 mm)			Deep pockets (≥6 mm)		
Treatment groups	Baseline	3 months	6 months	Baseline	3 months	6 months	Baseline	3 months	6 months	Baseline	3 months	6 months	Baseline	3 months	6 months	Baseline	3 months	6 months
Group 1 (SRP) n = 50	37.48 ± 6.9^Aa^	18.43 ± 4.4^Ab^	20.29 ± 7.1^Abc^	38.43 ± 7.3^Aa^	24.94 ± 5.2^Ab^	28.15 ± 6.7^Abc^	5.08 ± 0.58^Aa^	4.35 ± 0.67^Aab^	3.81 ± 0.85^Abc^	6.32 ± 0.77^Aa^	5.73 ± 0.97^Aab^	5.06 ± 1.13^Abc^	4.89 ± 0.93^Aa^	4.06 ± 1.03^Aab^	3.43 ± 1.01^Abc^	6.67 ± 0.71^Aa^	6.06 ± 0.94^Aab^	5.51 ± 0.79^Abc^
Group 2 (SRP+PDT) n = 50	33.86 ± 7.1^Aa^	11.49 ± 3.2^Bb^	14.61 ± 5.4^Bbc^	40.19 ± 8.2^Aa^	21.88 ± 6.8^Bb^	18.82 ± 5.1^Bc^	4.90 ± 0.61^Aa^	3.16 ± 0.73^Bb^	3.02 ± 0.58^Abc^	6.41 ± 1.03^Aa^	4.72 ± 0.84^Bb^	4.21 ± 0.59^Abc^	5.44 ± 0.93^Aa^	3.79 ± 0.96^Ab^	3.47 ± 1.03^Ab^c	6.83 ± 1.03Aa	5.27 ± 1.03^Bab^	4.86 ± 0.77^Bbc^
Group 3 (SPR+AV gel) n = 50	35.76 ± 5.59^Aa^	13.24 ± 3.7^BCb^	15.43 ± 6.1^BCc^	36.32 ± 4.9^Aa^	12.54 ± 3.2^Cb^	10.33 ± 3.3^Cbc^	4.97 ± 0.48^Aa^	4.25 ± 0.82^ACab^	3.54 ± 0.42^Abc^	6.19 ± 1.26^Aa^	5.29 ± 0.97^ACb^	5.04 ± 0.91^Abc^	4.57 ± 0.69^Aa^	3.47 ± 1.05^Ab^	3.12 ± 0.94^Abc^	6.91 ± 1.08^Aa^	5.90 ± 1.01^ABb^	5.19 ± 1.10^ABbc^

A−C: Different capital letters mean statistically significant difference at specific time point (baseline or 3rd month or 6th month) in individual clinical parameter between the treatment groups (p < 0.05). a−c: Different lower case letter means statistically significant difference for each clinical parameter recorded on follow-up (baseline or 3rd month or 6th month) for same treatment group (p < 0.05).

**Fig 1:**
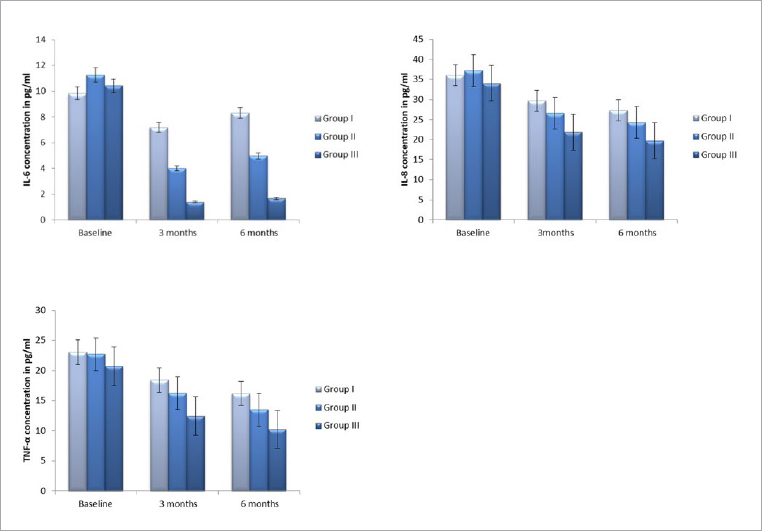
Graphic representation of changes in levels of cytokines in GCF (mean ± standard deviation) pre- and post- treatment at three time points (baseline/3 and 6 months) for three treatment groups 1. IL-6 levels; 2. IL-8 levels; and 3. TNF-α levels.

The levels of IL-6, IL-8 and TNF-α factor were identified for pre- and post- treatment for three groups at different time points (baseline/ 3 months / 6 months). A statistically significant reduction in IL-6 levels was observed for groups 2 and 3 in comparison to group 1 at the 3-month follow-up (p < 0.05; Fig 1a). Correspondingly, IL-8 (Fig 1b) and TNF-α (Fig 1c) showed statistically significant reduction for groups 2 and 3 in comparison to group 1. Further reduction in these two factors was observed at the 6-month follow-up visit.

## Discussion

Periodontitis is a multifactorial disease involving alterations between the microbial content of biofilm and a susceptible host.^17,24,27,40^ Most commonly, SRP is considered as the standard non-surgical treatment of choice for reducing PD inflammation. Because SRP unaccompanied by adjunct therapies has not been found to be successful in a majority of cases, adjunct therapies are used in combination with the standard treatment protocol.^17^ The effects of various chemical and herbal extracts have been evaluated in both in vivo and in vitro studies.^9,17,22,37^ The current study used a novel approach to evaluate and compare the effect of AV gel and PDT used as adjunct therapies to SRP in patients with chronic periodontitis. At the end of the study, the group treated with PDT+SRP displayed statistically significant improvement in PI, reduced PD pocket depth and improved CAL, whereas the group with AV gel intervention displayed reduction of the three inflammatory parameters (IL-6, IL-8 and TNF-α) in chronic periodontitis patients.

There is reliable evidence that short- and long-term use of photodynamic therapy (PDT) improves periodontal parameters in patients with chronic periodontitis.^5,7^ Although one study reported that PDT tended to improve all the periodontal parameters by the 3- and 6- month follow-up,^26^ Petelin et al^32^ found no statistically significant improvements in CAL and PD pocket depth with PDT.^32^ Thus, further clinical trials are required to resolve this controversy.

The effects of various adjunct topical medications on periodontitis have been evaluated. Antibiotics are considered the gold standard for treating periodontal infections, although several adverse effects such as allergic and gastrointestinal reactions can occur.^15,31^ Thus, in the present clinical trial, the effect of natural/herbal AV gel was evaluated to develop a medication for treating chronic periodontitis with minimal or no side effects.

AV gel contains 75 active components, including multiple vitamins, sugars, enzymes, lignins, saponins, salicylic and amino acids.^43^ The results of the current study are in agreement with Moghaddam et al,^6^ who found statistically significant improvement in PI and GI with the use of AV gel. It also helped reduce the PD pocket depth. Substantially reduced plaque scores in our study depended on effective prior manual plaque debridement with proper oral hygiene instructions.

In various in vitro and animal models, the main constituents M6PR (mannose 6-phosphate receptor), CA (calcium), GPx (glutathione peroxidase), and SOD (superoxide dismutase) in AV proved to have strong anti-inflammatory, anti-microbial and anti-oxidant effects.^18^ Thus, in our study, AV gel effectively helped reduce gingival bleeding. Biju et al^12^ demonstrated that the GPx and SOD components of AV gel help reduce the occurrence of PD diseases (gingivitis and periodontitis). Additionally, they demonstrated a statistically significant reduction in PD pocket depth.^12^

In chronic periodontitis, the pro-inflammatory factors involved in bone resorption include IL-6, IL-8 and TNF-α.^42^ In the present study, the treatment groups showed a slight increase these cytokines at the 6-month follow-up, which is in agreement with Niazi et al,^30^ who found similar changes in cytokine levels at follow-up.

To date, no clinical trial trials have been conducted to evaluate and compare the therapeutic modalities of AV gel and photosensitiser on chronic periodontitis. Additionally, in the present study, stratification of patients into moderate (4–5 mm) and severe (≥ 6 mm) periodontal pocket depths helped in better understanding topical therapeutic medications.

The limitations of this clinical trial included a relatively short follow-up period. Furthermore, the concentration of AV gel was not calibrated. Additionally, the anti-microbial potential of AV was not evaluated. Clinical trials with longer follow-up periods and microbiological analyses could help to devise improved therapeutic schemes to use AV extract as a medication.

## Conclusion

Photodynamic therapy and aloe vera gel used as a topical adjunct therapy for chronic periodontitis effectively reduces PD inflammation. Photodynamic therapy statistically significantly reduces PI, PD pocket depth and improves CAL, whereas AV gel statistically significantly reduced the scores of gingival bleeding.
